# Fabrication of Magnetic Poly(L-lactide) (PLLA)/Fe_3_O_4_ Composite Electrospun Fibers

**DOI:** 10.3390/ma17153773

**Published:** 2024-08-01

**Authors:** Zhu Liu, Yufu Zheng, Lizhong Lin, Xiaofei Liu, Na Qiang

**Affiliations:** 1 School of Materals Science and Engineering, Tianjing University, Tianjin 300350, China; lz890927@126.com (Z.L.); zhengyf@nbsds.com.cn (Y.Z.); 13095913015@163.com (L.L.); 2 Ningbo Sidson Vibration Reduction System Co., Ltd., Ningbo 315700, China; 3 Guangdong Provincial Education Department Development Team of Advanced Material Coating and Surface Interface Technology, Huizhou Engineering Technology Research Center of Advanced Coating Materials, Dayawan Chemical Engineering Research Institute, Huizhou University, Huizhou 516007, China; 4 School of Dayawan Chemical and New Materials, Huizhou University, Huizhou 516007, China; 5 School of Chemistry and Materials Engineering, Huizhou University, Huizhou 516007, China

**Keywords:** electrospinning, PLLA, Fe_3_O_4_ NPs, electrospinning conditions, magnetic property

## Abstract

Electrospinning technology is widely used for preparing biological tissue engineering scaffolds because of its advantages of simple preparation, accurate process parameters, and easy control. Poly(L-lactide) (PLLA) is regarded as a promising biomass-based polymer for use in electrospinning. The incorporation of Fe_3_O_4_ nanoparticles (NPs) could improve the osteogenic differentiation and proliferation of cells in the presence or absence of a static magnetic field (SMF). In this work, these two materials were blended together to obtain electrospun samples with better dispersibility and improved magnetic properties. First, composite PLLA and Fe_3_O_4_ NP fibers were prepared by means of electrospinning. The influence of electrospinning conditions on the morphology of the composite fibers was then discussed. Changes in magnetic properties and thermal stability resulting from the use of different PLLA/Fe_3_O_4_ mass ratios were also considered. Next, the morphology, crystal state, thermodynamic properties, and magnetic properties of the electrospun samples were determined using scanning electron microscopy (SEM), thermogravimetric analysis (TGA), differential scanning calorimetry (DSC), X-ray diffraction (XRD), Fourier-transform infrared spectroscopy (FTIR), and vibration sample magnetization (VSM). The results showed that the fibers prepared using PLLA with *M*_n_ = 170,000 exhibited good morphology when electrospun at 12 KV. The magnetic properties of PLLA/Fe_3_O_4_ composite electrospun fibers increased with the NP content, with the exception of thermal stability. The results of the present study may help to promote the further development of PLLA/Fe_3_O_4_ composite materials in the biomedical field.

## 1. Introduction

Bone injuries and defects remain a challenge in the field of clinical therapy. Bone tissue engineering is widely regarded as the most promising treatment method for diseased or damaged tissues [1,2]. The basic strategy of bone tissue engineering involves the development of bioactive implants to repair defective bone tissues. Bone tissue engineering is performed using porous scaffolds in combination with tissue cells and relative factors to assist cell growth [3,4]. This is most effectively achieved by mimicking the morphological traits and physical signals of the native bone extracellular matrix (ECM) [5,6,7].

The ECM plays an important role because it can control cell behavior. Nanofibrous scaffolds exhibit physical properties that are similar to those of the natural extracellular matrix, specifically adhesion, proliferation, and differentiation [8,9]. Electrospinning is widely used for nanofiber fabrication as it is a convenient and inexpensive production method [10,11,12]. Many synthetic polymers can be used for electrospinning; these include poly(ε-caprolactone) (PCL) [13,14], poly(lactic-co-glycolic acid) (PLGA) [15,16], and poly(L-lactic acid) (PLLA) [17]. The synthetic copolymers of L-lactide acid (L-LA) and ε-caprolactone (ε-CL) and L-LA and glycolic acid (GA) (i.e., PLCL and PLGA) are good scaffold candidates because of their biological compatibility [18,19,20]. PLLA is an environmentally friendly material that is obtained from natural resources and exhibits good biodegradability and biocompatibility [21,22]. It has been approved for biomedical applications by the U.S. Food and Drug Administration (FDA). Electrospun PLLA nanofiber has already been widely applied in bone tissue engineering [23,24].

In bone tissue engineering, scaffolds should be able to provide cues to stimulate osteoprogenitor or stem cells into forming an osteogenic phenotype. Physical signals such as magnetic signals have gradually begun to be applied to the field of bone tissue engineering, adding to the beneficial features of nanofibrous structures [25,26]. Magnetic nanoparticles (MNPs) can promote osteoblastic activity and bone formation. Currently, MNPs mainly include Fe_3_O_4_ particles with diameters of 10 and 20 nm. MNPs have been shown to increase the osteogenic differentiation of osteoblasts and stimulate osteoclast apoptosis [27,28]. The beneficial effects of nanofibrous structure and magnetic fields on bone regeneration have inspired the development of magnetic biodegradable fibrous materials. MNPs have usually been embedded in polymers for tissue engineering and drug release [29]. And polymer matrices have been widely used in tissue engineering [30,31]. For instance, an investigation of osteogenic differentiation effects in mesenchyme cells was carried out using graphene oxide–iron (II,III) oxide (GO-Fe_3_O_4_) composite, and it was found that GO-Fe_3_O_4_ noticeably increased osteogenic differentiation in bone marrow mesenchymal stem cells (BMSCs) [32]. In another study by Ma et al., silk fibroin (SF) and polyacrylic acid (PAA) were employed to modify Fe_3_O_4_ NPs. The results indicated that SF hydrogel containing Fe_3_O_4_@PAA NPs reduced intracellular ROS-induced damage and thus improved cell activity. Finally, the authors of the study found that the ALP activity, mineralization ability, and collagen secretion levels of MSCs on SF hydrogel with Fe_3_O_4_@PAA NPs were all higher under a magnetic field [33]. G C Li et al. prepared directionally aligned polycaprolactone/triiron tetraoxide (PCL/Fe_3_O_4_) fiber scaffolds by the electrospinning technique. And then they grafted them with the IKVAV peptide for regulating DRG growth and axon extension in peripheral nerve regeneration. The results showed that oriented aligned magnetic PCL/Fe_3_O_4_ composite scaffolds were successfully prepared by the electrospinning technique and possessed good mechanical properties and magnetic responsiveness [34].

In light of the above, a magnetic PLLA/Fe_3_O_4_ nanofibrous film was prepared in the present study. Firstly, PLLA was dissolved in hexafluoroisopropanol at different ratios. By controlling the variable parameters in the electrospinning process, the morphological characteristics of the obtained composite electrospun fibers were evaluated in order to obtain the highest-quality composite nanofiber. Secondly, PLLA/Fe_3_O_4_ electrospinning solutions with different proportions were prepared. Subsequently, the relevant properties were investigated by using thermal analysis, infrared spectroscopy, and X-ray diffraction analysis. Finally, the morphological characteristics and magnetic properties of the obtained composite electrospun fibers were evaluated. This study is expected to provide a theoretical and experimental basis for tissue engineering.

## 2. Materials and Methods

### 2.1. Materials

PLLA was purchased from Jinan Daigang Biomaterial Co., Ltd. (Jinan, China), Fe_3_O_4_ (20 nm) was purchased from Aladdin (Shanghai, China), Hexafluoroisopropanol was purchased from Shanghai Macklin Biochemical Co., Ltd. (Shanghai, China). The other reagents used in this experiment were purchased from Huizhou Nanyuan Chemical Products Co., Ltd. (Huizhou, China).

### 2.2. Characterization

SEM: The samples were sputter-coated with gold and visualized via scanning electron microscopy (SEM) (JSM-6380LA Analytical SEM, JEOL Ltd., Tokyo, Japan) operated at an accelerating voltage of 15 kV. The nanofibers’ diameter was a mean value of 100 filaments of each sample from different SEM images determined by using National Institutes of Health (NIH) Image J software (1.44P, Bethesda, MD, USA).

TGA: The thermal decomposition temperature was investigated using a thermogravimetric analyzer (TG 209 F1, Netzsch, Selb, Germany) purged with nitrogen. The analysis was carried out over a temperature range from 35 to 600 °C at a scanning rate of 10 °C/min and a N_2_ flow of 20 mL/min.

DSC: The crystallization behavior of the polymers was investigated using a modulated differential scanning calorimeter (MDSC 2910, TA Instruments, New Castle, DE, USA) purged with nitrogen. DSC was carried out over a temperature range from 0 °C to 250 °C at a scanning rate of 10 °C/min. 

XRD: the crystal structures of the samples were investigated using a X-ray powder diffractometer (Ultima IV, Rigaku, Tokyo, Japan) with Cu Kα radiation (λ = 1.5406 Å and 40 kV, 40 mA).

Vibration sample magnetization (VSM): M–H curves for PLLA/Fe_3_O_4_ NP composite electrospun fibers were evaluated by carrying out VSM (JDAW-2000D, Changchun Yingpu Magnetoelectric Technology Development Co., LT, Changchun, China) at room temperature.

### 2.3. Methods

First, a measured amount of PLLA was dissolved in hexafluoroisopropanol. The solution was stirred at room temperature for 12 h so that the PLLA was fully dissolved. Fe_3_O_4_ nanoparticles were then added to the fully dissolved PLLA solution. Next, the solution was ultrasonically shocked in an ice-water bath for 30 min so that the nanoparticles were evenly dispersed. Six batches of electrospinning fluid were configured under the above conditions, with composition and mass/volume ratios as shown in Table 1.

Stabilized electrospinning solution was then loaded into a syringe and placed on the work table of the injection system. Then, the injection block was moved so that it just made contact with the syringe handle. Both positive and negative high-voltage contacts were clamped, and the distance between the receiver and the needle was adjusted to the appropriate position (12 cm). Positive and negative high voltages were then adjusted to appropriate values. Next, the electrospinning solution was pulled to a cone under high static electricity, forming a Taylor cone. The electrospinning solution formed a jet, which was dispersed into a multi-strand jet under a strong electric field. Finally, the sample was attached to the surface and accumulated for electrospinning. The electrospinning time for each solution was 30 min. The flow rate value was 1 mL/h. To prevent Fe_3_O_4_ nanoparticles from settling, the solution containing Fe_3_O_4_ nanoparticles was removed after every 10 min of electrospinning and subjected to 2 min of ultrasonic shock before electrospinning was resumed.

The effects on electrospun fibers of variations in the PLLA molecular weight, electrospinning liquid concentration, electrospinning process factors, and ratio of PPLA to Fe_3_O_4_ are presented in Table 2, Table 3 and Table 4, which provide detailed electrospinning parameters, as follows.

## 3. Results and Discussion

### 3.1. Effect of Molecular Weight (PLLA) on Electrospun Fibers

The polymer solution concentration, molecular weight, and viscosity are critical factors that affect short fiber size [34,35]. When the molecular weight was low, the interaction between polymer chains was insufficient. The electrospinning was affected because it was difficult for stable polymer fibers to form. Other influencing factors included solubility and the interactions of polymer chains. When the solubility of a low-molecular-weight polymer in organic solvents was high, it was difficult for continuous fibers to form; fibers broke during electrospinning, and the morphology of fibers was also affected. Moreover, when the molecular weight was low, the interactions of polymer chains were weak, so that it was difficult for a stable polymer solution to be formed.

**Figure 1:**
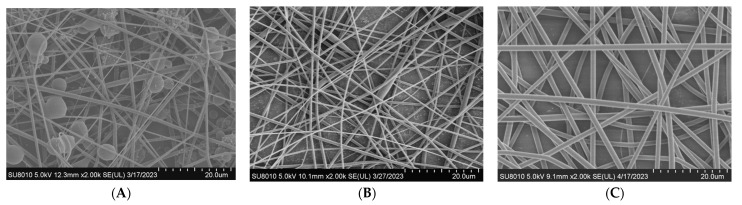
SEM images of PLLA with different molecular weights. (**A**) Molecular weight of PLLA (*M*_n_ = 20,000). (**B**) Molecular weight of PLLA (*M*_n_ = 83,000). (**C**) Molecular weight of PLLA (*M*_n_ = 170,000).

The electrospun fibers of PLLA (*M*_n_ = 20,000) exhibited large numbers of spheres (Figure 1). The fiber diameter was very uneven, and some fibers were even fractured. In addition, the spinning jet was presented in a fog. When the molecular weight was 83,000, the fibers still had a spindle shape, but fiber diameter was still very uneven. When the molecular weight increased to 170,000, the fibers were smoother and more uniform, the diameters of the fibers increased, and the spinning jet presented a better umbrella shape. In light of these findings, subsequent experiments were carried out using the molecular weight *M*_n_ = 170,000 to explore other factors.

### 3.2. Effect of Polymer Concentration on Diameters of Electrospun Fibers

**Figure 2:**
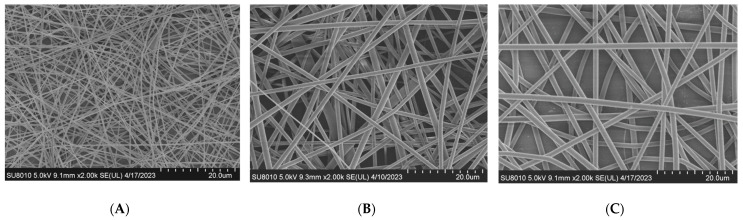
SEM image of PLLA with different solution concentrations. (**A**) Solution concentration 6%. (**B**) Solution concentration 9%. (**C**) Solution concentration 12%.

**Figure 3:**
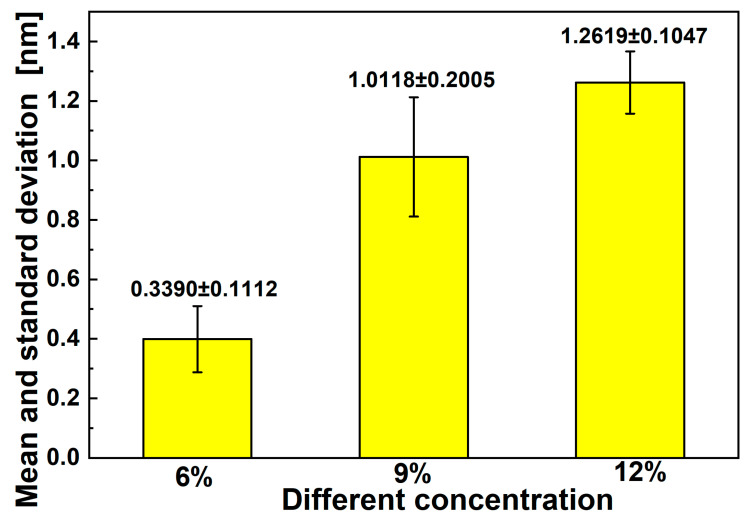
Means and standard deviations of fiber diameters for different concentrations of solution.

As shown in Figure 2 and Figure 3, when other conditions remained unchanged, the diameters of electrospun fibers increased as the solution concentration increased. The mean diameter of electrospun fibers increased from 400 nm to 1200 nm as the concentration of the solution increased from 6% to 12%. This is because the viscosity of the solution also increased with an increase in concentration. When the voltage remained unchanged during the electrospinning process, the electrostatic field force also remained unchanged. When the viscosity was higher, more electrospinning materials were contained in the thin stream into which the solution jet was dispersed, and a coarser single-spun bundle was finally formed. There were spindles in the fiber when the concentration was 6%; however, these disappeared as the concentration increased. This was because the solution was prone to melt rupture under the action of injection pressure. Put simply, it was not easy for fiber structures to form when the concentration was lower than a certain value. When the concentration was 12%, fibers did not exhibit spindles and their diameter was more uniform. Subsequent experiments for exploring other factors were therefore carried out at a concentration of 12%.

### 3.3. Effect of Electrospinning Mode on Diameter of Fibers

**Figure 4:**
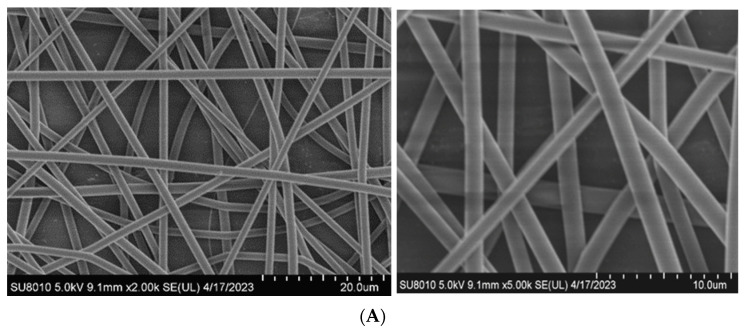

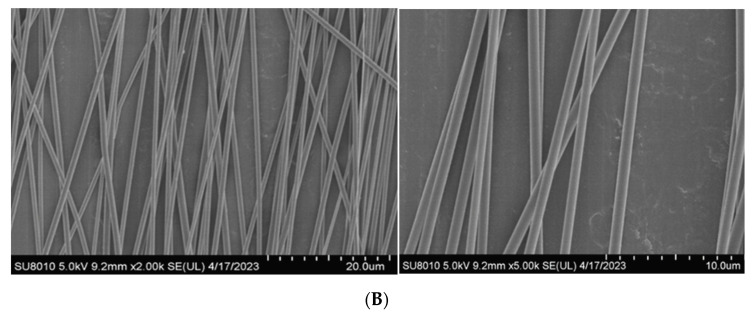
SEM images of fibers produced using different spinning processes. (**A**) Random electrospinning. (**B**) Oriented electrospinning.

**Figure 5:**
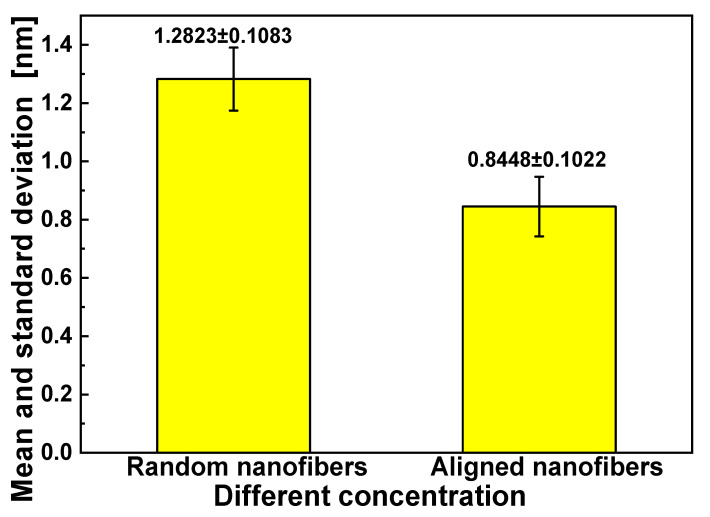
Means and standard deviations of fiber diameters for different collectors.

As presented in Figure 4 (*M*_n_ = 17,000, 12%) and Figure 5, the uniformity in the diameters of electrospun fibers produced using an oriented spinning process did not differ greatly from that when a random spinning process was used. However, the mean diameter increased from 830 nm to 1280 nm when the collector was changed from aligned electrospun fibers to random nanofibers. The fiber diameter of the oriented group was lower than that of the random group. This was because the high-speed rotation of the orientation receiver caused electrospun fibers to be subjected to mechanical tensile forces in addition to electric forces. These stronger forces caused the fibers to be stretched thinner. However, there was no obvious difference in the fiber diameter with different MNP concentrations, indicating that the addition of MNP did not affect the fiber diameter.

### 3.4. Thermogravimetric Analysis (TGA)

**Figure 6:**
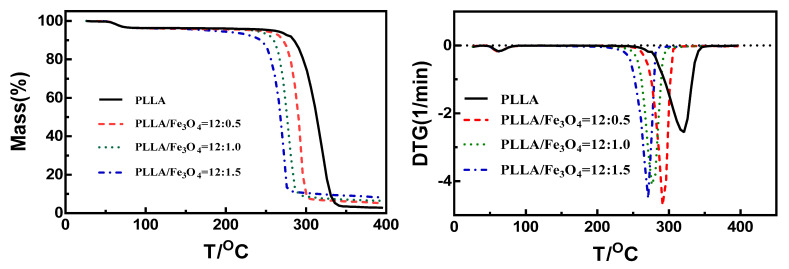
TGA and DTG curves of PLLA/Fe_3_O_4_ electrospun fibers.

The 5% weight loss temperature is usually used as a measure of thermal stability. In the present study, we found almost no difference between the change trends of the TGA curves for the PLLA and PLLA/Fe_3_O_4_ composite electrospun fibers. There were two stages to the thermal decomposition process. The first certain mass loss during initial heating was due to the evaporation of physical adsorption water. The second stage involved the decomposition of blended fiber products. From Figure 6 and Table 5, it can be seen that the initial decomposition temperature of the sample decreased from 266.2 °C to 232.8 °C after doping with Fe_3_O_4_, and the decomposition rate also accelerated significantly. The thermogravimetric initial temperature and the end temperature of the fiber both decreased with increased Fe_3_O_4_ content. The thermal decomposition stability of PLLA composite electrospun fiber was weakened by the addition of Fe_3_O_4_ [36,37]. This may have been due to the high surface area and chemical activity of Fe_3_O_4_ nanoparticles. Furthermore, the maximum decomposition temperature decreased from 346.2 °C to 286.8 °C when the ratio of PLLA/Fe_3_O_4_ changed from 12:0 to 12:1.5. This could have been because the thermal decomposition reaction of PLLA fiber was catalyzed by Fe_3_O_4_ nanoparticles in the thermal decomposition process. Because Fe_3_O_4_ nanoparticles exhibit high levels of chemical stability and thermal catalytic activity, their addition was bound to accelerate the thermal decomposition process.

### 3.5. Differential Scanning Calorimetry (DSC)

**Figure 7:**
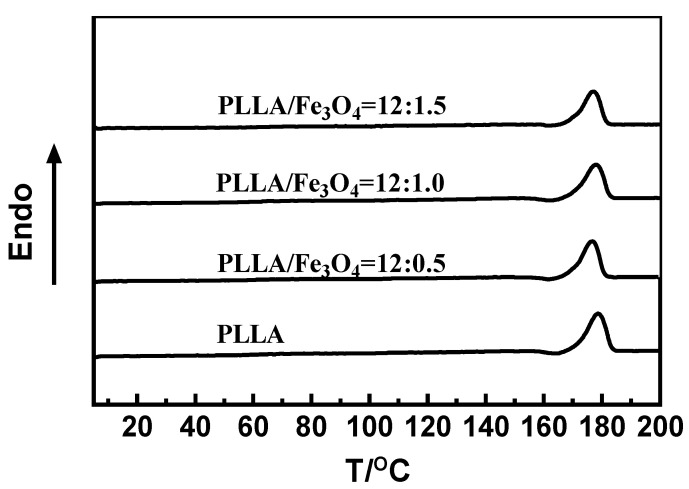
DSC curve of PLLA/Fe_3_O_4_ composite electrospun fibers.

The melting temperature and crystallization were obtained by means of DSC. DSC curves of electrospun samples with different ratios of PLLA/Fe_3_O_4_ are presented in Figure 7. It can be seen that the melting temperature of the samples did not change significantly. It was also determined that the addition of Fe_3_O_4_ nanoparticles basically did not change the crystallization condition of electrospun samples.

### 3.6. Infrared Spectroscopic Analysis (FTIR)

PLLA/Fe_3_O_4_ NPs were characterized using FTIR to confirm their chemical compositions (Figure S1). FTIR spectra over the range 4000~500 cm^−1^ for electrospun fibers are presented in Figure S1. The spectra showed stretching vibration in the C–H bond of PLLA methylene at 2950 cm^−1^. The characteristic bands at 456 cm^−1^ and 1380 cm^−1^ indicated asymmetric and symmetrical bending vibrations of methyl groups in PLLA. The absorption peak at 1130 cm^−1^ indicated the rocking vibrations of methyl groups in PLLA. The absorption peaks at 1047 cm^−1^ and 870 cm^−1^ corresponded to C–CH_3_ and C–COO, respectively. A comparison of the IR spectra revealed no significant differences between pure PLLA and PLLA/Fe_3_O_4_ electrospun fibers. However, in the case of the latter, there was no characteristic peak at 890 cm^−1^ of Fe_3_O_4_ because the number of Fe_3_O_4_ nanoparticles was too small, and infrared absorption was weak. It appears that the FTIR spectrum of PLLA/Fe_3_O_4_ NPs was a combination of pure Fe_3_O_4_ NPs and PLLA. There was no chemical bond between the nanoparticles and the electrospun fibers.

### 3.7. X-ray Analysis (XRD)

**Figure 8:**
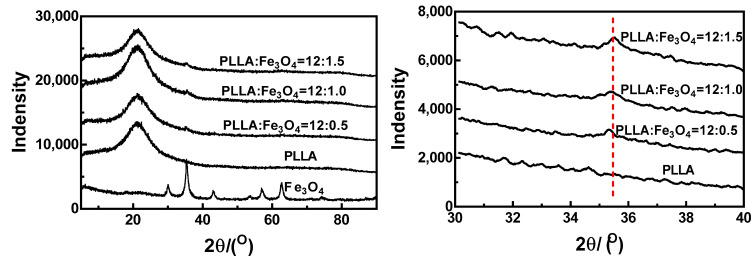
XRD curves for PLLA/Fe_3_O_4_ composite electrospun fibers.

XRD curves for PLLA/Fe_3_O_4_ composite electrospun fibers are shown in Figure 8. The crystal diffraction peak of PLLA was at around 2θ = 21.8°, and this peak did not change significantly with increased Fe_3_O_4_ content. This indicated that the addition of Fe_3_O_4_ nanoparticles did not affect the formation of PLLA crystals. A local magnification of 2θ = 30°~40° is presented in the right-hand figure. The characteristic peak of Fe_3_O_4_ in the PLLA/Fe_3_O_4_ composite fiber appeared at about 35.4°, and this was enhanced with increased Fe_3_O_4_ content. These findings demonstrated the presence of Fe_3_O_4_ nanoparticles in this composite system.

### 3.8. Magnetic Analysis

**Figure 9:**
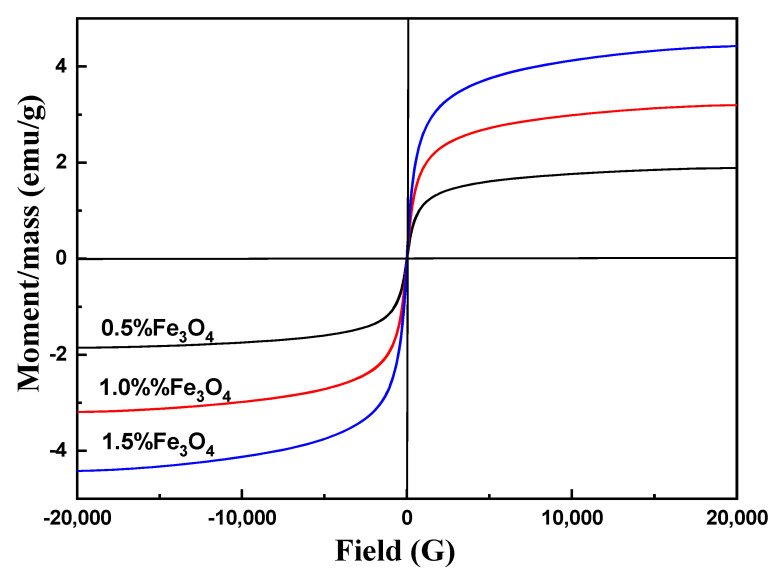
VSM analysis of PLLA/Fe_3_O_4_ composite electrospun fibers.

VSM (vibrating sample magnetometer) analysis was the primary method to study the magnetic properties of materials. The result of this analysis can be used to calculate data such as saturation magnetization magnetic permeability and to identify the magnetic classification of the material such as Ferro, para, and superparamagnetic. The VSM image of PLLA/Fe_3_O_4_ is shown in Figure 9. According to Figure 9, in the range of 0–20,000 Oe, the induced magnetic property in the sample increased with the increasing external magnetic field, and the maximum amount of magnetization in the PLLA reached 4.42 emu/g. Saturation magnetization was achieved at 1.88 emu/g, 3.19 emu/g, and 4.42 emu/g when the Fe_3_O_4_ ratios were 0.5%, 1.0%, and 1.5%, respectively. In addition, the magnetic properties of PLLA/Fe_3_O_4_ composite electrospun fibers increased with increasing Fe_3_O_4_ content. The degree of magnetization reached zero when the strength of the external magnetic field became zero. These results indicated that the nanoparticles and composite nanomaterials exhibited superparamagnetic characteristics.

## 4. Conclusions

Many reports have discussed the effect of magnetic fields on bone regeneration and reconstruction. Magnetic scaffolds may be prepared by introducing Fe_3_O_4_ NPs into biodegradable polymers. In this work, magnetic PLLA/Fe_3_O_4_ composite electrospinning scaffolds were prepared. Firstly, the influencing factors in the electrospinning process were discussed, such as the molecular weight, the concentration of electrospinning solution, and the type of electrospinning collector used. We found that it was difficult to form stable polymer fibers when the molecular weight and solution concentration were low because the interchain interaction forces acting on the polymer were weak. We also found that, under the same electrospinning conditions, uniformity in the diameters of the fibers was significantly affected by the orientation process used. However, the diameters of the electrospun fibers were smaller. Subsequently, the effect of the addition of Fe_3_O_4_ was analyzed using FTIR, XRD, DSC, and TGA. This analysis revealed that chemical structure was not affected after the addition of Fe_3_O_4_, but the thermal stability decreased. We also found that the electrospun fibers exhibited good magnetic response performance, and the magnetism increased with increasing Fe_3_O_4_ NP content. In the present study, scaffolds with the advantageous features of fiber uniformity and magnetism were prepared. This result has the potential for extensive use in the fabrication of scaffolds for bone tissue engineering.

## Figures and Tables

**Table 1:** Compositions and mass/volume ratios of different batches of electrospinning solutions.

Number	Composition (Mass Ratio)	Mass/Volume (*w*/*v*%)
1	PLLA/Fe_3_O_4_ = 12:0	6
2	PLLA/Fe_3_O_4_ = 12:0	9
3	PLLA/Fe_3_O_4_ = 12:0	12
4	PLLA/Fe_3_O_4_ = 12:0.5	12
5	PLLA/Fe_3_O_4_ = 12:1	12
6	PLLA/Fe_3_O_4_ = 12:1.5	12

**Table 2:** Molecular weights of PLLA.

Number	Composition	Mass/Volume (*w*/*v*%)	Voltage (kV)	*M*_n_
1	PLLA	12	10	20,000
2	PLLA	12	10	83,000
3	PLLA	12	10	170,000

**Table 3:** Concentrations of electrospinning solution.

Number	Composition	Mass/Volume (*w*/*v*%)	Voltage (kV)	Collector Type
1	PLLA	8	10	random electrospun fibers
2	PLLA	10	10	random electrospun fibers
3	PLLA	12	10	random electrospun fibers

**Table 4:** Electrospinning processes of PLLA.

Number	Composition	Mass/Volume (*w*/*v*%)	Voltage (kV)	Collector Type
1	PLLA	12	12	random
2	PLLA	12	12	orientation

**Table 5:** Decomposition temperature of samples.

Samples	Thermal Decomposition Temperature (°C)	Maximum Decomposition Temperature (°C)
PLLA	266.2	346.2
PLLA/Fe_3_O_4_ = 12:0.5	252.1	307.1
PLLA/Fe_3_O_4_ = 12:1	246.9	294.9
PLLA/Fe_3_O_4_ = 12:1.5	232.8	286.8

## Data Availability

The raw data supporting the conclusions of this article will be made available by the authors on request.

## Supplementary Materials

The following supporting information can be downloaded at: https://www.mdpi.com/article/10.3390/ma17153773/s1, Figure S1: Infrared curve of PLLA/Fe_3_O_4_ composite electrospinning nanofibers.

## References

[1] Meng C., Tang D., Liu X., Meng J., Wei W., Gong R.H., Li J. (2023). Heterogeneous porous PLLA/PCL fibrous scaffold for bone tissue regeneration. Int. J. Biol. Macromol..

[2] Habibzadeh F., Sadraei S.M., Mansoori R., Singh Chauhan N.P., Sargazi G. (2022). Nanomaterials supported by polymers for tissue engineering applications: A review. Heliyon.

[3] Kotlarz M., Melo P., Ferreira A.M., Gentile P., Dalgarno K. (2023). Cell seeding via bioprinted hydrogels supports cell migration into porous apatite-wollastonite bioceramic scaffolds for bone tissue engineering. Biomater. Adv..

[4] González S.G., Vlad M.D., López J.L., Aguado E.F. (2023). Novel bio-inspired 3D porous scaffold intended for bone-tissue engineering: Design and in silico characterisation of histomorphometric, mechanical and mass-transport properties. Mater. Desig..

[5] Serrano-Aroca A., Cano-Vicent A., Sabater I.S.R., El-Tanani M., Aljabali A., Tambuwala M.M., Mishra Y.K. (2022). Scaffolds in the microbial resistant era: Fabrication, materials, properties and tissue engineering applications. Mater. Today Bio.

[6] Entz L., Falgayrac G., Chauveau C., Pasquier G., Lucas S. (2022). The extracellular matrix of human bone marrow adipocytes and glucose concentration differentially alter mineralization quality without impairing osteoblastogenesis. Bone Rep..

[7] Seo Lee J., Nah H., Lee D., An S.-H., Ko W.-K., Jin Lee S., Nyoung Heo D. (2022). Immediately implantable extracellular matrix-enriched osteoinductive hydrogel-laden 3D-printed scaffold for promoting vascularized bone regeneration in vivo. Mater. Des..

[8] da Silva D.M., Barroca N., Pinto S.C., Semitela Â., de Sousa B.M., Martins P.A.D., Marques P.A.A.P. (2023). Decellularized extracellular matrix-based 3D nanofibrous scaffolds functionalized with polydopamine-reduced graphene oxide for neural tissue engineering. Chem. Eng. J..

[9] Feng B., Ji T., Wang X., Fu W., Ye L., Zhang H., Li F. (2020). Engineering cartilage tissue based on cartilage-derived extracellular matrix cECM/PCL hybrid nanofibrous scaffold. Mater. Des..

[10] Luraghi A., Peri F., Moroni L. (2021). Electrospinning for drug delivery applications: A review. J. Control Release.

[11] Hauser M., Nowack B. (2021). Probabilistic modelling of nanobiomaterial release from medical applications into the environment. Environ. Int..

[12] Lian S., Lamprou D., Zhao M. (2024). Electrospinning technologies for the delivery of Biopharmaceuticals: Current status and future trends. Int. J. Pharm..

[13] Bhattacharjee P., Madden P.W., Patriarca E., Ahearne M. (2023). Optimization and evaluation of oxygen-plasma-modified, aligned, poly (capital JE, Ukrainian- caprolactone) and silk fibroin nanofibrous scaffold for corneal stromal regeneration. Biomater. Biosyst..

[14] Dorati R., Conti B., Colzani B., Dondi D., Lazzaroni S., Modena T., Genta I. (2018). Ivermectin controlled release implants based on poly-D, l -lactide and poly-ε-caprolactone. J. Drug Deliv. Sci. Technol..

[15] Bahcecioglu G., Hasirci N., Hasirci V. (2019). Cell behavior on the alginate-coated PLLA/PLGA scaffolds. Int. J. Biol. Macromol..

[16] Qiao T., Jiang S., Song P., Song X., Liu Q., Wang L., Chen X. (2016). Effect of blending HA-g-PLLA on xanthohumol-loaded PLGA fiber membrane. Colloids Surf. B Biointerfaces.

[17] Zahir L., Kida T., Tanaka R., Nakayama Y., Shiono T., Kawasaki N., Nakayama A. (2020). Synthesis and properties of biodegradable thermoplastic elastomers using 2-Methyl-1,3-propanediol, succinic acid and lactide. Polym. Degrad. Stab..

[18] Radwan N.H., Nasr M., Ishak R.A.H., Awad G.A.S. (2021). Moxifloxacin-loaded in situ synthesized Bioceramic/Poly(L-lactide-co-epsilon-caprolactone) composite scaffolds for treatment of osteomyelitis and orthopedic regeneration. Int. J. Pharm..

[19] Qiang N., Lin W., Zhou X., Liu Z., Lu M., Qiu S., Tang S., Zhu J. (2021). Electrospun fibers derived from peptide coupled amphiphilic copolymers for dorsal root ganglion (DRG) outgrowth. Gels.

[20] Tang Y., Chen L., Zhao K., Wu Z., Wang Y., Tan Q. (2016). Fabrication of PLGA/HA (core)-collagen/amoxicillin (shell) nanofiber membranes through coaxial electrospinning for guided tissue regeneration. Compos. Sci. Technol..

[21] Momeni P., Nourisefat M., Farzaneh A., Shahrousvand M., Abdi M.H. (2024). The engineering, drug release, and in vitro evaluations of the PLLA/HPC/Calendula Officinalis electrospun nanofibers optimized by Response Surface Methodology. Heliyon.

[22] Frydlova B., Fajstavr D., Slepickova Kasalkova N., Rimpelova S., Svobodova Pavlickova V., Svorcik V., Slepicka P. (2023). Replicated biopolymer pattern on PLLA-Ag basis with an excellent antibacterial response. Heliyon.

[23] Estrada R.G., Multigner M., Fagali N., Lozano R.M., Munoz M., Cifuentes S.C., Lieblich M. (2023). Metastable FeMg particles for controlling degradation rate, mechanical properties, and biocompatibility of Poly(l-lactic) acid (PLLA) for orthopedic applications. Heliyon.

[24] Chen Y., Shafiq M., Liu M., Morsi Y., Mo X. (2020). Advanced fabrication for electrospun three-dimensional nanofiber aerogels and scaffolds. Bioact. Mater..

[25] Li W., Zeng G., Yan J., Liu X., Jiang X., Yang J., Sun D. (2021). One-pot green synthesis of I@CNDs-Fe_3_O_4_ hybrid nanoparticles from kelp for multi-modal imaging in vivo. Mater. Sci. Eng. C Mater. Biol. Appl..

[26] Lewinska A., Adamczyk-Grochala J., Bloniarz D., Olszowka J., Kulpa-Greszta M., Litwinienko G., Pazik R. (2020). AMPK-mediated senolytic and senostatic activity of quercetin surface functionalized Fe_3_O_4_ nanoparticles during oxidant-induced senescence in human fibroblasts. Redox Biol..

[27] Niu Z., Murakonda G.K., Jarubula R., Dai M. (2021). Fabrication of Graphene oxide-Fe_3_O_4_ nanocomposites for application in bone regeneration and treatment of leukemia. J. Drug Deliv. Sci. Technol..

[28] Ren R., Guo J., Song H., Wei Y., Luo C., Zhang Y., Xiong W. (2023). A novel implant surface modification mode of Fe_3_O_4_-containing TiO_2_ nanorods with sinusoidal electromagnetic field for osteoblastogenesis and angiogenesis. Mater. Today Bio.

[29] Komlev A.S., Gimaev R.R., Zverev V.I. (2021). Smart magnetocaloric coatings for implants: Controlled drug release for targeted delivery. Phys. Open.

[30] Kubota M., Yokoi T., Ogawa T., Saito S., Furuya M., Yokota K., Kawashita M. (2021). In-vitro heat-generating and apatite-forming abilities of PMMA bone cement containing TiO_2_ and Fe_3_O_4_. Ceram. Int..

[31] Huan W., Dong M., Li J., Xie M., Huang Y., Carlini R., Yang Y. (2023). Magnetic properties of Al^3+^ doped Fe_3_O_4_ by solid-solid reaction and biological applications of GQDs grafted MNPs. J. Magn. Magn. Mater..

[32] Baladi M., Amiri M., Salavati-Niasari M. (2023). Green sol–gel auto-combustion synthesis, characterization and study of cytotoxicity and anticancer activity of ErFeO_3_/Fe_3_O_4_/rGO nanocomposite. Arab. J. Chem..

[33] Ma Y., Yang J., Hu Y., Xia Z., Cai K. (2022). Osteogenic differentiation of the MSCs on silk fibroin hydrogel loaded Fe_3_O_4_@PAA NPs in static magnetic field environment. Colloids Surf. B Biointerfaces.

[34] Ura D.P., Stachewicz U. (2024). Direct electrospinning of short polymer fibers: Factors affecting size and quality. Compos. Part A Appl. Sci. Manuf..

[35] Fathona I.W., Yabuki A. (2014). A simple one-step fabrication of short polymer nanofibers via electrospinning. J. Mater. Sci..

[36] Navarro Oliva F.S., Sahihi M., Lenglet L., Ospina A., Guenin E., Jaramillo-Botero A., Goddard W.A., Bedoui F. (2023). Nanoparticle size and surface chemistry effects on mechanical and physical properties of nano-reinforced polymers: The case of PVDF-Fe_3_O_4_ nano-composites. Polym. Test..

[37] Hingrajiya R.D., Patel M.P. (2023). Fe_3_O_4_ modified chitosan based co-polymeric magnetic composite hydrogel: Synthesis, characterization and evaluation for the removal of methylene blue from aqueous solutions. Int. J. Biol. Macromol..

