# Supplementary material for: Fabrication of Magnetic Poly(L-lactide) (PLLA)/Fe3O4 Composite Electrospun Fibers
Source: Materials (Basel). 2024 Aug 1;17(15):3773. doi: 10.3390/ma17153773 (PMC11312587; doi:10.3390/ma17153773)
Supplement: Supplementary file 1 [file materials-17-03773-s001.zip › materials-2988525-supplementary.pdf]

Supporting Information

Fabrication of Magnetic Poly(L-lactide) (PLLA)/Fe_3O_4 Composite Electrospun Fibers

Zhu Liu [1,2,3,4,5], Yufu Zheng [1,2], Lizhong Lin [1,2], Xiaofei Liu [1,2,*] and Na Qiang [1,3,4,5,*]

1 School of Materals Science and Engineering, Tianjing University, Tianjin 300350, China
2 Ningbo Sidson Vibration Reduction System Co., Ltd., Ningbo 315700, China
3 Guangdong Provincial Education Department Development Team of Advanced Material Coating and Sur face Interface Technology, Huizhou Engineering Technology Research Center of Advanced Coating Materials, Dayawan Chemical Engineering Research Institute, Huizhou University, Huizhou 516007, China
4 School of Dayawan Chemical and New Materials, Huizhou University, Huizhou 516007, China
5 School of Chemistry and Materials Engineering, Huizhou University, Huizhou 516007, China
* Correspondence: liuxf@tju.edu.cn (X.L.); qiangna93@163.com (N.Q.)

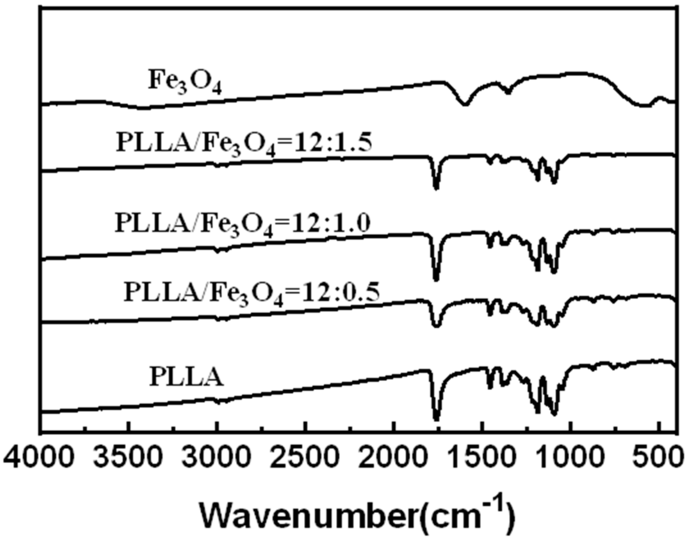

Figure S1. Infrared curve of PLLA/Fe_3O_4 composite electrospinning nanofibers.
